# Progress in Biomaterials and Technologies in Dentistry

**DOI:** 10.3390/biomedicines12071482

**Published:** 2024-07-04

**Authors:** Giuseppe Minervini

**Affiliations:** 1 Saveetha Institute of Medical and Technical Sciences (SIMATS), Saveetha Dental College and Hospitals, Saveetha University, Chennai 00202, Tamil Nadu, India; giuseppe.minervini@unicampania.it; 2 Multidisciplinary Department of Medical-Surgical and Dental Specialties, University of Campania Luigi Vanvitelli, 98201 Naples, Italy

The field of dental biomedicine continues to evolve with significant advancements that are aimed at improving oral health outcomes. This Special Issue discusses the state-of-the-art findings that are of importance for future clinical practices and treatment methodologies. Further, consideration has been given to reports on the latest developments relating to the wider stage of dental research, in order to give the reader a full panorama of the current situation. One such recent study looked into the potential of casein phosphopeptide-amorphous calcium phosphate in remineralizing molar–incisor hypomineralized teeth; the authors used polarized Raman and scanning electron microscopy to document the structural changes in hypomineralized enamel after a 28-day treatment protocol [1]. Following this methodology, the authors found considerable improvements in the mineral density and organization of the enamel crystals in the white and yellow MIH lesions, reaching approximately two-thirds of their original values in the lesions that became remineralized [1,2]. Therefore, this line of research supports an increase in the use of CPP-ACP in clinical practice in order to increase the mechanical properties of teeth that are affected by MIH, reducing the susceptibility of these teeth to abrasion and carious lesions for both white and yellow MIH lesions [1]. This work details the general advances in the field in terms of the applications for CPP-ACP in dentistry [3,4]. Among the important effects, silver diamine fluoride (SDF) has received wide acclaim for its antibacterial properties and in its prevention of carious lesion progression [5,6,7]. A systematic review in the recent literature evaluates the impact of SDF on bacterial biofilms. The meta-analysis included data obtained from 15 studies that showed the success of SDF in reducing biofilm formation by the general bacterial species Streptococcus mutans and Actinomyces naeslundii. One of the studies proves the hypothesis that SDF, aside from the inhibition of biofilm development, has a preferred odds ratio of 3.59 and a risk ratio of 1.63 compared to other groups of antibacterial agents, thus indicating its potential for being the standard treatment of dental caries and its allied ailments [8,9,10,11]. The application of CPP-ACP for the remineralization of MIH teeth offers a promising non-invasive treatment option that can enhance enamel integrity and reduce hypersensitivity [1]. Similarly, SDF’s role in biofilm management presents a cost-effective and efficient solution that is particularly beneficial in community health settings where access to traditional dental care may be limited [5,6]. However, there are areas that warrant further investigation [12]. The long-term effects and durability of both CPP-ACP and SDF treatments need to be explored through extended clinical trials. Additionally, standardized protocols for the application of these treatments would enhance their reproducibility and reliability in clinical settings.

Recent studies have also aimed to explore other innovative approaches in dental care. For example, research on the use of Er:YAG laser-induced pre-emptive pulpal laser analgesia (PPLA) has demonstrated promising results in reducing enamel surface alterations [13,14]. The study found that using specific laser parameters could minimize the damage to enamel during dental procedures, offering a potential non-pharmacological alternative for pain management [13,14,15]. This technique could be particularly useful in pediatric dentistry, where minimizing patient discomfort is paramount.

In addition, another important finding of the latest studies concerns the assessment of the remineralization of these agents above the enamel. A study directly compared the effect of various remineralizing agents, such as fluoride varnishes, with biomimetic hydroxyapatite in the restoration of enamel integrity [16,17]. The results obtained corresponded to those where, while fluoride is a gold standard, the efficacy of biomimetic hydroxyapatite was comparable, suggesting the latter as an alternative to the gold standard treatment for this problem [16,17]. It may be quite important if the patient expresses some sensitivities or contraindications to fluoride.

In materials science, bioinorganic technology has led to the introduction of new composites and bonding agents not only resulting in better strength and esthetic results, but also advances in material research that have led to the mechanization of these materials; they can mimic the mechanical properties of the natural tooth structure, thereby increasing their functionality in restorative dentistry. Recently, the effect of different orthodontic treatments on periodontal health has also been studied in an orthodontic setting [18,19]. There has been a longitudinal study with respect to the effects of fixed and removable orthodontic appliances on changes in gingival inflammation and plaque accumulation within the oral cavity [19,20,21]. Evidence indicates that, while both appliances functioned in an effective manner to achieve the same tooth alignment purpose, it was possible that there might be few benefits of removable appliances for the better maintenance of periodontal health; this is due to the easy cleaning and maintenance of these appliances [19,22]. These results have been consistent with those of other community studies, which have also assessed the effects of such an appliance on general health.

This Special Issue aims to explore the role of digital technology in enhancing dental diagnostics and treatment planning. The use of digital impressions and 3D printing has changed prosthodontics dramatically by making dental prostheses much more accurate and patient-specific [23,24]. It has significantly improved the inaccuracies associated with dental restorations; at the same time, the patient was more satisfied due to a decrease in chair time and discomfort from traditional impressions.

Recently, interprofessional collaboration has emerged as a very important agent in the development of dental research and practice [25]. These studies underpin the way in which interdisciplinary approaches may be necessary for the solution of complex problems in the area of dental health; they also present examples of how efforts to align dental researchers and microbiologists assist in the explanation of the microbial dynamics of oral biofilm, a mechanism of targeted antibacterial therapy [25]. Indeed, these cooperative efforts underscore a larger trend in integrated community research approaches to elevate dental health outcomes.

The inclusion of patient-centered research is a noteworthy aspect of recent advancements in dental care. Studies focusing on patient-reported outcomes and satisfaction provide valuable insights into the effectiveness of various dental treatments from the patient’s perspective. This approach ensures that patient needs and preferences are at the forefront of clinical decision-making, ultimately leading to improved treatment experiences and outcomes. These findings align with a broader movement in the dental research community towards prioritizing patient-centered care.

Within recent years, current advances in diagnostic imaging are hyperlinked to a dramatic rise in the ability to detect and diagnose dental diseases [26,27]. Among imaging techniques, such as high-resolution digital imaging, entropy measurements are applied to improve the quality of radiographs, thus also raising the possibility of identifying radiographic signs of dental diseases, including the EOTRH syndrome (equine odontoclastic tooth resorption and hypercementosis).

In addition, a study on the efficacy of desensitizing toothpastes provides new insights into managing dentin hypersensitivity (DH) [28,29]. The research evaluated three commercial toothpastes for their ability to occlude dentinal tubules and enhance dentin hardness. The results indicated the significant occlusion of dentinal tubules by all tested toothpastes, with products containing nano-hydroxyapatite and NovaMin technology showing increased calcium and phosphorus content on the dentin surface [30]. However, none of the toothpastes significantly increased dentin hardness, highlighting the importance of selecting appropriate desensitizing agents for DH management [29].

Further exploring the effects of rapid palatal expansion (RPE) on oral tissues, another study examined the clinical and histopathological changes in gingival and palatal mucosa during RPE treatment [31,32]. The research highlighted that the type of palatal expander influenced the degree of inflammation, with mini-implant-anchored expanders causing more pronounced hyperplasia compared to tooth-borne expanders [33]. This provides valuable insights into optimizing orthodontic treatments to minimize adverse effects on soft oral tissues. The integration of digital technologies in orthodontics has also been a focal point in recent research. Digital tools such as 3D imaging and printing are transforming treatment planning and execution, enabling more precise and personalized orthodontic solutions [34,35]. These technologies not only improve clinical outcomes but also enhance patient comfort and satisfaction by reducing the invasiveness and duration of treatments.

## References

[1] Cardoso-Martins I., Pessanha S., Coelho A., Arantes-Oliveira S., Marques P.F. (2022). Evaluation of the Efficacy of CPP-ACP Remineralizing Mousse in Molar-Incisor Hypomineralized Teeth Using Polarized Raman and Scanning Electron Microscopy—An In Vitro Study. Biomedicines.

[2] Yassaei S., Aghili H., Shahraki N., Safari I. (2018). Efficacy of Erbium-Doped Yttrium Aluminum Garnet Laser with Casein Phosphopeptide Amorphous Calcium Phosphate with and without Fluoride for Remineralization of White Spot Lesions around Orthodontic Brackets. Eur. J. Dent..

[3] Minervini G., Franco R., Marrapodi M.M., Di Blasio M., Isola G., Cicciù M. (2023). Conservative Treatment of Temporomandibular Joint Condylar Fractures: A Systematic Review Conducted According to PRISMA Guidelines and the Cochrane Handbook for Systematic Reviews of Interventions. J. Oral. Rehabil..

[4] Minervini G., Franco R., Marrapodi M.M., Di Blasio M., Ronsivalle V., Cicciù M. (2023). Children Oral Health and Parents Education Status: A Cross Sectional Study. BMC Oral Health.

[5] Zhao I.S., Gao S.S., Hiraishi N., Burrow M.F., Duangthip D., Mei M.L., Lo E.C.-M., Chu C.-H. (2018). Mechanisms of Silver Diamine Fluoride on Arresting Caries: A Literature Review. Int. Dent. J..

[6] Zhang J., Got S.-R., Yin I.X., Lo E.C.-M., Chu C.-H. (2021). A Concise Review of Silver Diamine Fluoride on Oral Biofilm. Appl. Sci..

[7] Mei M.L., Chu C.H., Low K.H., Che C.M., Lo E.C.M. (2013). Caries Arresting Effect of Silver Diamine Fluoride on Dentine Carious Lesion with S. Mutans and L. Acidophilus Dual-Species Cariogenic Biofilm. Med. Oral. Patol. Oral. Cir. Bucal.

[8] Mubaraki H., Ingle N.A., Baseer M.A., Al Mugeiren O.M., Mubaraki S., Cicciù M., Minervini G. (2023). Effect of Silver Diamine Fluoride on Bacterial Biofilms—A Review Including In Vitro and In Vivo Studies. Biomedicines.

[9] Alkahtany M., Beatty M.W., Alsalleeh F., Petro T.M., Simetich B., Zhou Y., Feely D., Polyzois G. (2023). Color Stability, Physical Properties and Antifungal Effects of ZrO2 Additions to Experimental Maxillofacial Silicones: Comparisons with TiO_2_. Prosthesis.

[10] Pelivan I., Šeparović I., Vuletić M., Dulčić N., Gabrić D. (2023). Radiological and Periodontal Evaluation of Stock and Custom CAD/CAM Implant Abutments—A One-Year Follow-Up Study. Prosthesis.

[11] Choi S., Kang Y.S., Yeo I.-S.L. (2023). Influence of Implant–Abutment Connection Biomechanics on Biological Response: A Literature Review on Interfaces between Implants and Abutments of Titanium and Zirconia. Prosthesis.

[12] Di Stasio D., Romano A., Paparella R.S., Gentile C., Serpico R., Minervini G., Candotto V., Laino L. (2018). How Social Media Meet Patients Questions: YouTube Review for Mouth Sores in Children. J. Biol. Regul. Homeost. Agents.

[13] Belcheva A., Veneva E., Hanna R. (2023). Effect of Various Protocols of Pre-Emptive Pulpal Laser Analgesia on Enamel Surface Morphology Using Scanning Electron Microscopy: An Ex Vivo Study. Biomedicines.

[14] Veneva E., Raycheva R., Belcheva A. (2018). Efficacy of Erbium-Doped Yttrium Aluminium Garnet for Achieving Pre-Emptive Dental Laser Analgesia in Children. Medicine.

[15] Goettems M.L., Zborowski E.J., Costa F.d.S., Costa V.P.P., Torriani D.D. (2017). Nonpharmacologic Intervention on the Prevention of Pain and Anxiety During Pediatric Dental Care: A Systematic Review. Acad. Pediatr..

[16] Lata S., Varghese N., Varughese J. (2010). Remineralization Potential of Fluoride and Amorphous Calcium Phosphate-Casein Phospho Peptide on Enamel Lesions: An in Vitro Comparative Evaluation. J. Conserv. Dent..

[17] Roveri N., Battistella E., Bianchi C.L., Foltran I., Foresti E., Iafisco M., Lelli M., Naldoni A., Palazzo B., Rimondini L. (2009). Surface Enamel Remineralization: Biomimetic Apatite Nanocrystals and Fluoride Ions Different Effects. J. Nanomater..

[18] Wu Y., Cao L., Cong J. (2020). The Periodontal Status of Removable Appliances vs Fixed Appliances. Medicine.

[19] Lucchese A., Bondemark L., Marcolina M., Manuelli M. (2018). Changes in Oral Microbiota Due to Orthodontic Appliances: A Systematic Review. J. Oral. Microbiol..

[20] Guo R., Lin Y., Zheng Y., Li W. (2017). The Microbial Changes in Subgingival Plaques of Orthodontic Patients: A Systematic Review and Meta-Analysis of Clinical Trials. BMC Oral Health.

[21] Liu H., Sun J., Dong Y., Lu H., Zhou H., Hansen B.F., Song X. (2011). Periodontal Health and Relative Quantity of Subgingival Porphyromonas Gingivalis during Orthodontic Treatment. Angle Orthod..

[22] Azaripour A., Weusmann J., Mahmoodi B., Peppas D., Gerhold-Ay A., Van Noorden C.J.F., Willershausen B. (2015). Braces versus Invisalign^®^: Gingival Parameters and Patients’ Satisfaction during Treatment: A Cross-Sectional Study. BMC Oral Health.

[23] Revilla-León M., Raney L., Piedra-Cascón W., Barrington J., Zandinejad A., Özcan M. (2020). Digital Workflow for an Esthetic Rehabilitation Using a Facial and Intraoral Scanner and an Additive Manufactured Silicone Index: A Dental Technique. J. Prosthet. Dent..

[24] Alqahtani J., Alhemaid G., Alqahtani H., Abughandar A., AlSaadi R., Algarni I., AlSharif W., Al-Harbi S., Burwaih R., Hasan A. (2022). Digital Diagnostics and Orthodontic Practice. J. Healthc. Sci..

[25] Luo T.L., Vanek M.E., Gonzalez-Cabezas C., Marrs C.F., Foxman B., Rickard A.H. (2022). In Vitro Model Systems for Exploring Oral Biofilms: From Single-species Populations to Complex Multi-species Communities. J. Appl. Microbiol..

[26] Kaasalainen T., Ekholm M., Siiskonen T., Kortesniemi M. (2021). Dental Cone Beam CT: An Updated Review. Phys. Medica.

[27] Di Nardo D., Zanza A., Pagnoni F., Xhajanka E., Testarelli L. (2022). An Update on Advanced Diagnostic Imaging in Dentistry. Diagnostics.

[28] Arnold W.H., Prange M., Naumova E.A. (2015). Effectiveness of Various Toothpastes on Dentine Tubule Occlusion. J. Dent..

[29] West N.X., Seong J., Davies M. (2015). Management of Dentine Hypersensitivity: Efficacy of Professionally and Self-administered Agents. J. Clin. Periodontol..

[30] Mahmoodi B., Goggin P., Fowler C., Cook R.B. (2021). Quantitative Assessment of Dentine Mineralization and Tubule Occlusion by NovaMin and Stannous Fluoride Using Serial Block Face Scanning Electron Microscopy. J. Biomed. Mater. Res. B Appl. Biomater..

[31] Tavares A., Braga E., Neves F.S. (2023). Influence of the Palatal Plane Cant and Skeletal Patterns in the Hard Palate Thickness?. Orthod. Craniofac Res..

[32] Bertucci V., Stevens K., Sidhu N., Suri S., Bressmann T. (2023). The Impact of Fan-Type Rapid Palatal Expanders on Speech in Patients with Unilateral Cleft Lip and Palate. Cleft Palate Craniofacial J..

[33] Kim K., Oh S., Kim B., Kim S. (2019). Asymmetric Nasomaxillary Expansion Induced by Tooth-bone-borne Expander Producing Differential Craniofacial Changes. Orthod. Craniofac. Res..

[34] Sangalli L., Savoldi F., Dalessandri D., Bonetti S., Gu M., Signoroni A., Paganelli C. (2021). Effects of Remote Digital Monitoring on Oral Hygiene of Orthodontic Patients: A Prospective Study. BMC Oral Health.

[35] Frazier-Bowers S.A., Allareddy V., Rengasamy Venugopalan S., Lamani E., Vora S.R., Kapila S. (2023). Preface to the 9th Biennial COAST Conference: Harnessing Technology and Biomedicine for Personalized Orthodontics. Orthod. Craniofac. Res..

